# Supplementary material for: Prognostic impact of catheter ablation in patients with asymptomatic atrial fibrillation
Source: PLoS One. 2022 Dec 15;17(12):e0279178. doi: 10.1371/journal.pone.0279178 (PMC9754597; doi:10.1371/journal.pone.0279178)
Supplement: S1 Fig — A) Event free rate from recurrent atrial tachyarrhythmias with a blanking period of 90 days after procedure. B) Discontinuation of OAC. AF = atrial fibrillation; OAC = oral anticoagulation. (PPTX) [file pone.0279178.s001.pptx]

## Slide 1
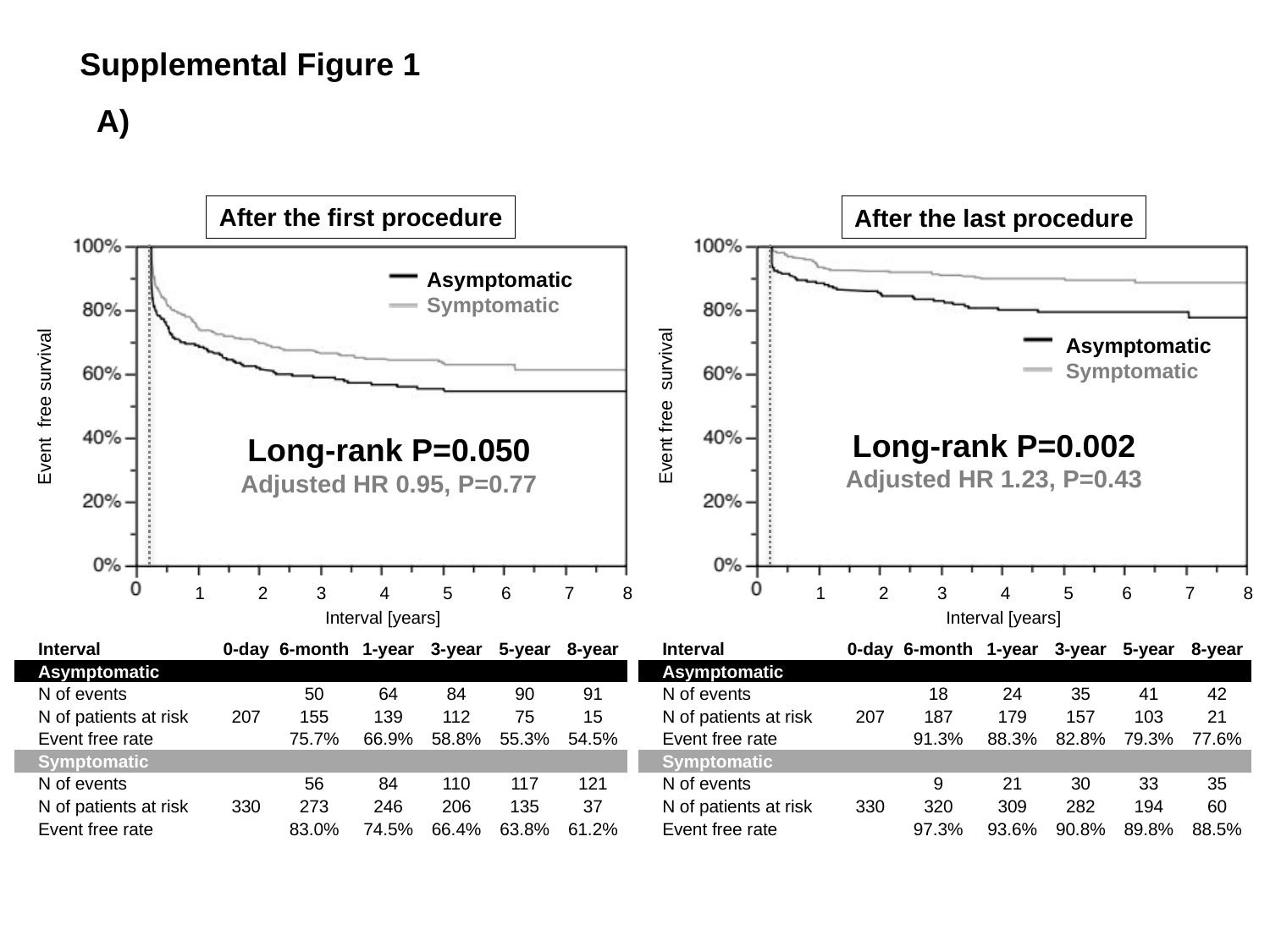

Supplemental Figure 1
A)
After the first procedure
After the last procedure
Asymptomatic
Symptomatic
Asymptomatic
Symptomatic
Event free survival
Event free survival
Long-rank P=0.002
Adjusted HR 1.23, P=0.43
Long-rank P=0.050
Adjusted HR 0.95, P=0.77
 1 2 3 4 5 6 7 8
 1 2 3 4 5 6 7 8
Interval [years]
Interval [years]
| Interval | 0-day | 6-month | 1-year | 3-year | 5-year | 8-year |
| --- | --- | --- | --- | --- | --- | --- |
| Asymptomatic | | | | | | |
| N of events | | 50 | 64 | 84 | 90 | 91 |
| N of patients at risk | 207 | 155 | 139 | 112 | 75 | 15 |
| Event free rate | | 75.7% | 66.9% | 58.8% | 55.3% | 54.5% |
| Symptomatic | | | | | | |
| N of events | | 56 | 84 | 110 | 117 | 121 |
| N of patients at risk | 330 | 273 | 246 | 206 | 135 | 37 |
| Event free rate | | 83.0% | 74.5% | 66.4% | 63.8% | 61.2% |
| Interval | 0-day | 6-month | 1-year | 3-year | 5-year | 8-year |
| --- | --- | --- | --- | --- | --- | --- |
| Asymptomatic | | | | | | |
| N of events | | 18 | 24 | 35 | 41 | 42 |
| N of patients at risk | 207 | 187 | 179 | 157 | 103 | 21 |
| Event free rate | | 91.3% | 88.3% | 82.8% | 79.3% | 77.6% |
| Symptomatic | | | | | | |
| N of events | | 9 | 21 | 30 | 33 | 35 |
| N of patients at risk | 330 | 320 | 309 | 282 | 194 | 60 |
| Event free rate | | 97.3% | 93.6% | 90.8% | 89.8% | 88.5% |

## Slide 2
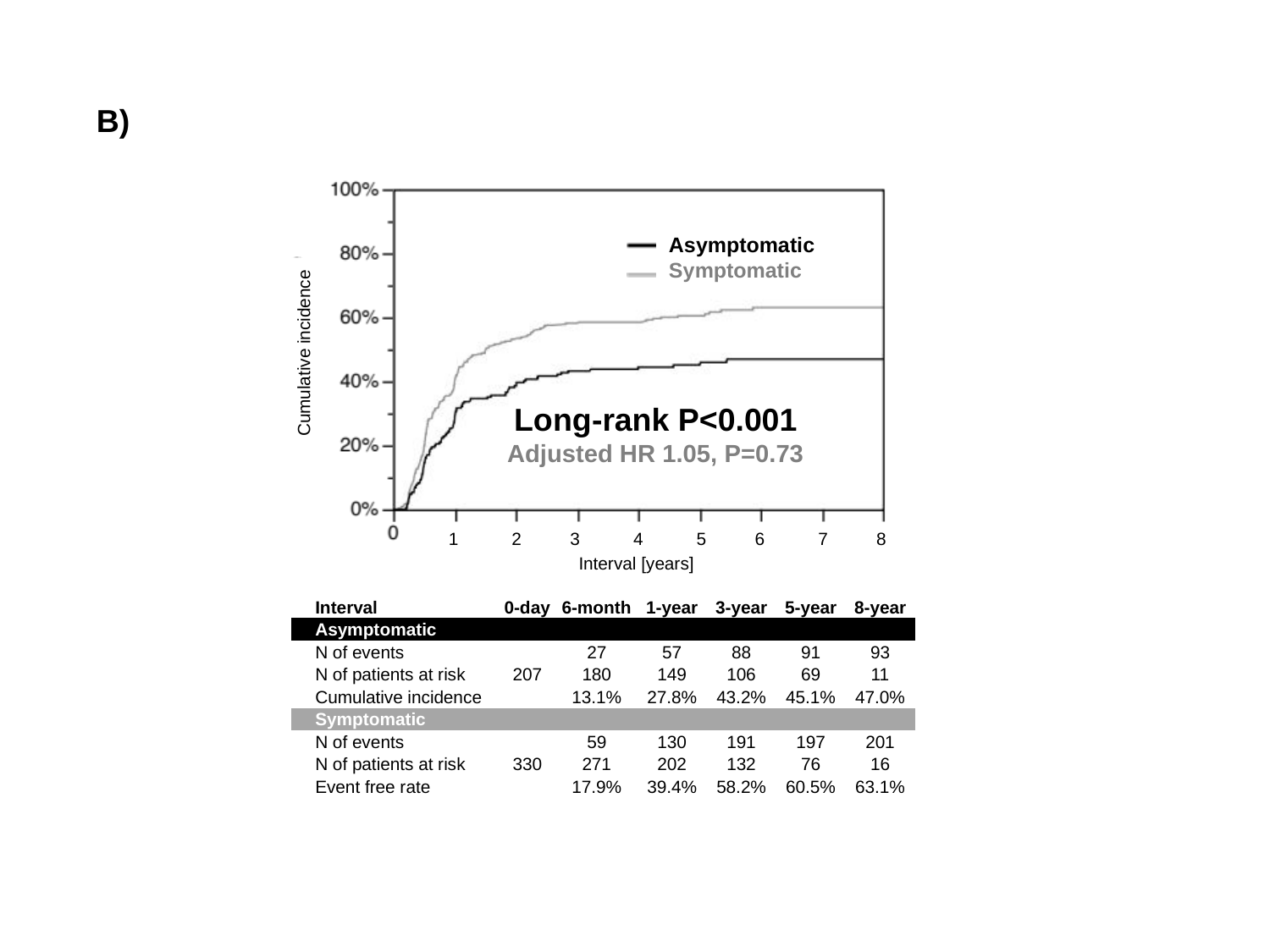

B)
Asymptomatic
Symptomatic
Cumulative incidence
Long-rank P<0.001
Adjusted HR 1.05, P=0.73
 1 2 3 4 5 6 7 8
Interval [years]
| Interval | 0-day | 6-month | 1-year | 3-year | 5-year | 8-year |
| --- | --- | --- | --- | --- | --- | --- |
| Asymptomatic | | | | | | |
| N of events | | 27 | 57 | 88 | 91 | 93 |
| N of patients at risk | 207 | 180 | 149 | 106 | 69 | 11 |
| Cumulative incidence | | 13.1% | 27.8% | 43.2% | 45.1% | 47.0% |
| Symptomatic | | | | | | |
| N of events | | 59 | 130 | 191 | 197 | 201 |
| N of patients at risk | 330 | 271 | 202 | 132 | 76 | 16 |
| Event free rate | | 17.9% | 39.4% | 58.2% | 60.5% | 63.1% |
