# Supplementary material for: Prognostic impact of catheter ablation in patients with asymptomatic atrial fibrillation
Source: PLoS One. 2022 Dec 15;17(12):e0279178. doi: 10.1371/journal.pone.0279178 (PMC9754597; doi:10.1371/journal.pone.0279178)
Supplement: S2 Fig — A) Event free rate from recurrent atrial tachyarrhythmias with a blanking period of 90 days after procedure. B) Discontinuation of OAC. AF = atrial fibrillation; OAC = oral anticoagulation. (PPTX) [file pone.0279178.s002.pptx]

## Slide 1
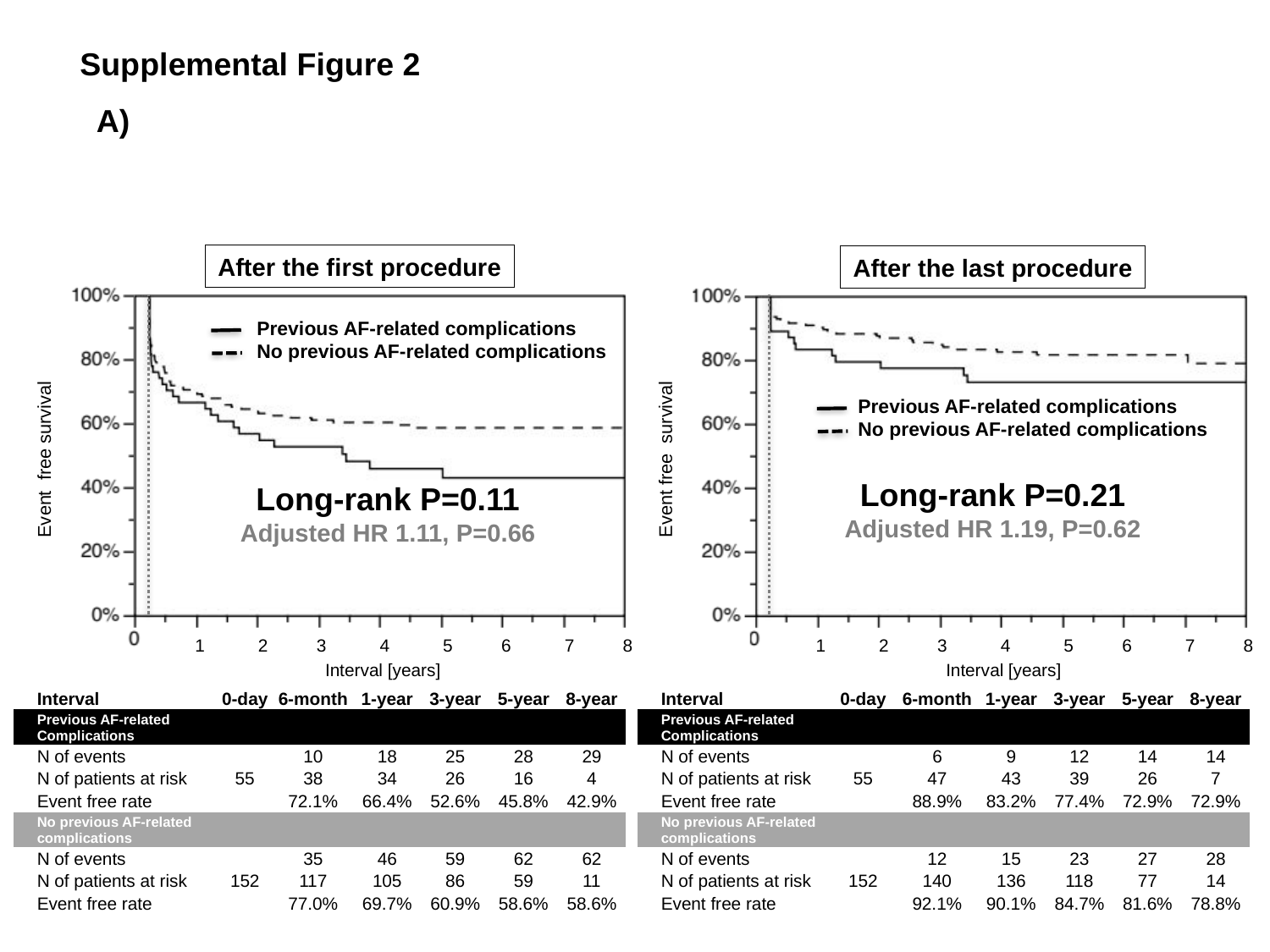

Supplemental Figure 2
A)
After the first procedure
After the last procedure
Previous AF-related complications
No previous AF-related complications
Previous AF-related complications
No previous AF-related complications
Event free survival
Event free survival
Long-rank P=0.21
Adjusted HR 1.19, P=0.62
Long-rank P=0.11
Adjusted HR 1.11, P=0.66
 1 2 3 4 5 6 7 8
 1 2 3 4 5 6 7 8
Interval [years]
Interval [years]
| Interval | 0-day | 6-month | 1-year | 3-year | 5-year | 8-year |
| --- | --- | --- | --- | --- | --- | --- |
| Previous AF-related Complications | | | | | | |
| N of events | | 10 | 18 | 25 | 28 | 29 |
| N of patients at risk | 55 | 38 | 34 | 26 | 16 | 4 |
| Event free rate | | 72.1% | 66.4% | 52.6% | 45.8% | 42.9% |
| No previous AF-related complications | | | | | | |
| N of events | | 35 | 46 | 59 | 62 | 62 |
| N of patients at risk | 152 | 117 | 105 | 86 | 59 | 11 |
| Event free rate | | 77.0% | 69.7% | 60.9% | 58.6% | 58.6% |
| Interval | 0-day | 6-month | 1-year | 3-year | 5-year | 8-year |
| --- | --- | --- | --- | --- | --- | --- |
| Previous AF-related Complications | | | | | | |
| N of events | | 6 | 9 | 12 | 14 | 14 |
| N of patients at risk | 55 | 47 | 43 | 39 | 26 | 7 |
| Event free rate | | 88.9% | 83.2% | 77.4% | 72.9% | 72.9% |
| No previous AF-related complications | | | | | | |
| N of events | | 12 | 15 | 23 | 27 | 28 |
| N of patients at risk | 152 | 140 | 136 | 118 | 77 | 14 |
| Event free rate | | 92.1% | 90.1% | 84.7% | 81.6% | 78.8% |

## Slide 2
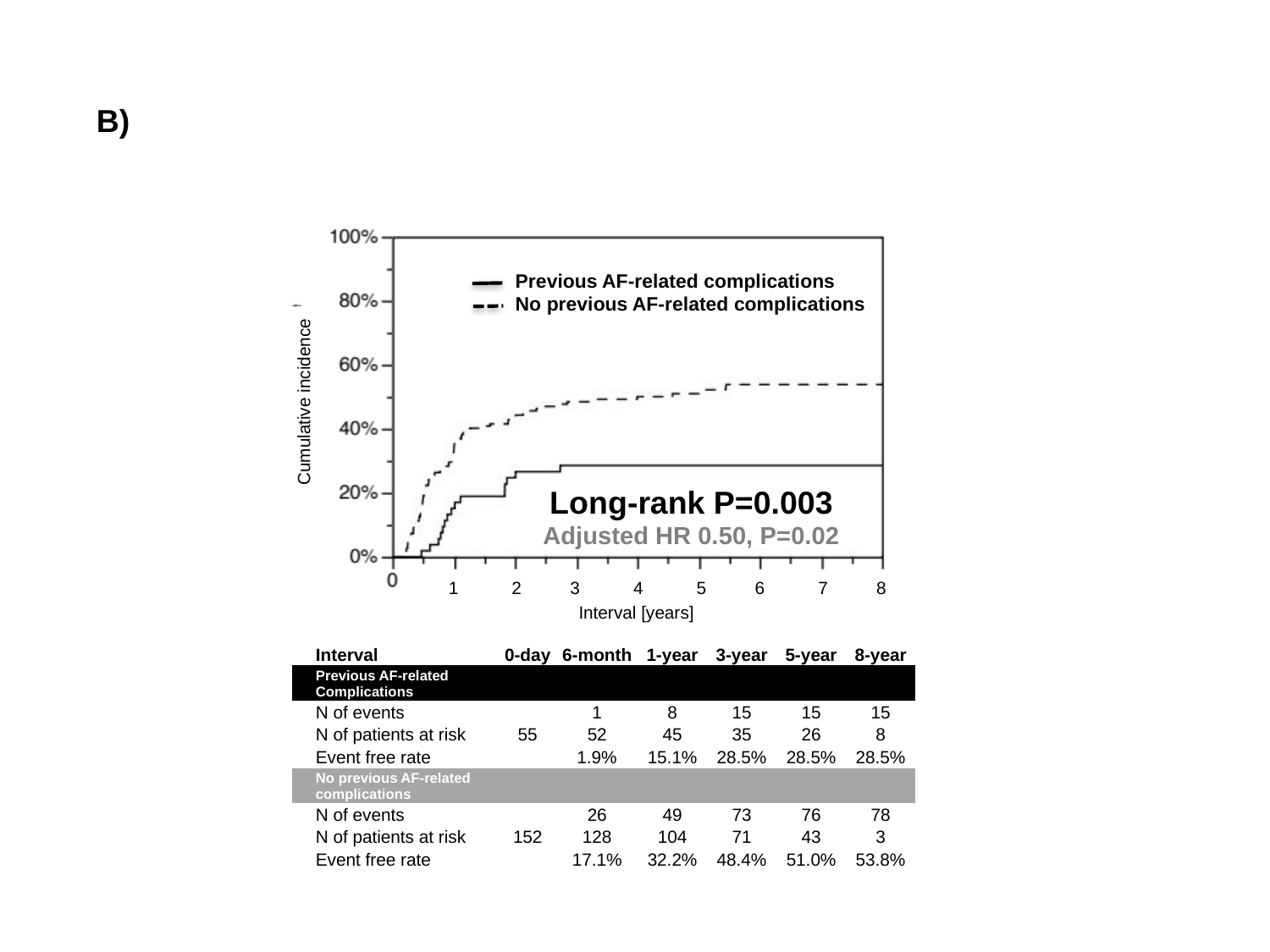

B)
Previous AF-related complications
No previous AF-related complications
Cumulative incidence
Long-rank P=0.003
Adjusted HR 0.50, P=0.02
 1 2 3 4 5 6 7 8
Interval [years]
| Interval | 0-day | 6-month | 1-year | 3-year | 5-year | 8-year |
| --- | --- | --- | --- | --- | --- | --- |
| Previous AF-related Complications | | | | | | |
| N of events | | 1 | 8 | 15 | 15 | 15 |
| N of patients at risk | 55 | 52 | 45 | 35 | 26 | 8 |
| Event free rate | | 1.9% | 15.1% | 28.5% | 28.5% | 28.5% |
| No previous AF-related complications | | | | | | |
| N of events | | 26 | 49 | 73 | 76 | 78 |
| N of patients at risk | 152 | 128 | 104 | 71 | 43 | 3 |
| Event free rate | | 17.1% | 32.2% | 48.4% | 51.0% | 53.8% |
