# Supplementary material for: Prognostic impact of catheter ablation in patients with asymptomatic atrial fibrillation
Source: PLoS One. 2022 Dec 15;17(12):e0279178. doi: 10.1371/journal.pone.0279178 (PMC9754597; doi:10.1371/journal.pone.0279178)
Supplement: S1 Table — (DOCX) [file pone.0279178.s003.docx]

**S1 Table: Details of ablation procedure**

|  | **Ablation group**  **N=537** |
| --- | --- |
| Pulmonary vein isolation | 537 (100%) |
| RFCA | 513 (95.6%) |
| Cryoballoon | 24 (4.4%) |
| Superior vena cava isolation | 144 (26.8%) |
| Complex fractionated atrial electrogram guided ablation | 152 (28.3%) |
| Tricuspid valve isthmus linear ablation | 509 (94.8%) |
| Mitral valve isthmus linear ablation | 14 (2.6%) |
| Non-pulmonary vein foci ablation | 7 (1.3%) |

RFCA=radiofrequency catheter ablation.
