# Supplementary material for: Prognostic impact of catheter ablation in patients with asymptomatic atrial fibrillation
Source: PLoS One. 2022 Dec 15;17(12):e0279178. doi: 10.1371/journal.pone.0279178 (PMC9754597; doi:10.1371/journal.pone.0279178)
Supplement: S2 Table — (DOCX) [file pone.0279178.s004.docx]

**S2 Table: Baseline characteristics according to AF symptom grades**

| **Baseline characteristics** | **Asymptomatic** | **Symptomatic** | | **P value** |
| --- | --- | --- | --- | --- |
|  | **EHRA**  **Grade 1**  **N=414** | **EHRA**  **Grade 2**  **N=542** | **EHRA**  **Grade 3 or 4**  **N=118** |  |
| Age (years old) | 66.2±6.8 | 65.9±8.6 | 68.5±9.3 | 0.005 |
| ≥ 75 years old | 41 (9.9%) | 77 (14.2%) | 38 (32.2%) | <0.001 |
| Women | 64 (15.5%) | 186 (34.3%) | 38 (32.2%) | <0.001 |
| Weight (kg) | 64.9±12.2 | 62.2±12.7 | 59.9±12.6 | <0.001 |
| Low body weight | 71 (18.1%) | 118 (23.1%) | 30 (27.0%) | 0.06 |
| Non-paroxysmal AF | 228 (56.0%) | 90 (16.6%) | 14 (11.9%) | <0.001 |
| AF duration (years) | 1.3 (0.4-4.5) | 1.4 (0.4-3.7) | 1.6 (0.4-4.2) | 0.73 |
| Hypertension | 283 (68.4%) | 338 (62.4%) | 71 (60.2%) | 0.09 |
| Diabetes | 83 (20.1%) | 100 (18.5%) | 15 (12.7%) | 0.17 |
| Previous AF-related complications | 97 (23.4%) | 59 (10.9%) | 17 (14.4%) | <0.001 |
| History of heart failure hospitalization | 37 (8.9%) | 12 (2.2%) | 9 (7.6%) | <0.001 |
| Ischemic stroke | 67 (16.2%) | 49 (9.0%) | 11 (9.3%) | 0.003 |
| CHA_2_DS_2_-VASc score | 2.3±1.5 | 2.2±1.5 | 2.2±1.5 | 0.25 |
| ≥ 2 | 267 (64.5%) | 358 (66.1%) | 81 (68.6%) | 0.69 |
| History of malignancy | 73 (17.6%) | 96 (17.7%) | 14 (17.0%) | 0.26 |
| eGFR (ml/min/1.73m^2^) | 51.3±16.3 | 55.0±17.3 | 51.2±16.3 | 0.002 |
| ≤60 ml/min/1.73m^2^ | 309 (76.7%) | 342 (65.5%) | 77 (69.4%) | 0.001 |
| **Echocardiographic data** |  |  |  |  |
| Left ventricular ejection fraction (%) | 62.1±12.5 | 66.0±10.3 | 67.1±10.1 | <0.001 |
| ≤ 40 % | 26 (6.9%) | 12 (2.3%) | 1 (0.9%) | <0.001 |
| Left atrial diameter (mm) | 43.4±8.3 | 39.5±7.2 | 39.6±8.0 | <0.001 |
| ≥ 50 mm | 68 (18.2%) | 48 (9.4%) | 12 (10.7%) | <0.001 |
| **Medications** |  |  |  |  |
| Oral anticoagulat | 376 (90.8%) | 453 (83.6%) | 96 (81.4%) | 0.001 |
| Warfarin | 208 (50.2%) | 234 (43.2%) | 50 (42.4%) | 0.07 |
| Direct oral anticoagulants | 168 (40.6%) | 221 (40.8%) | 46 (39.0%) | 0.94 |
| Antiplatelet use | 93 (22.5%) | 104 (19.2%) | 26 (22.0%) | 0.44 |
| Anti-arrhythmic drugs | 76 (18.4%) | 198 (36.5%) | 40 (33.9%) | <0.001 |
| Beta blockers | 145 (35.0%) | 167 (30.8%) | 47 (39.8%) | 0.12 |
| Verapamil/diltiazem | 52 (12.6%) | 81 (14.9%) | 24 (20.3%) | 0.12 |
| Digitalis | 51 (12.3%) | 46 (8.5%) | 12 (10.2%) | 0.15 |
| ACEI/ARB | 191 (46.1%) | 210 (38.8%) | 45 (38.1%) | 0.05 |

Categorical variables are presented as number (percentage). Continuous variables are presented as mean ± SD or median and interquartile range.

AF=atrial fibrillation; BNP=brain natriuretic peptide; eGFR=estimated glomerular filtration rate; EHRA=European Heart Rhythm Association.
