# Supplementary material for: Prognostic impact of catheter ablation in patients with asymptomatic atrial fibrillation
Source: PLoS One. 2022 Dec 15;17(12):e0279178. doi: 10.1371/journal.pone.0279178 (PMC9754597; doi:10.1371/journal.pone.0279178)
Supplement: S3 Table — (DOCX) [file pone.0279178.s005.docx]

**S3 Table: Baseline characteristics of asymptomatic and symptomatic AF patients with and without previous AF-related complications**

|  | **Asymptomatic**  **No previous**  **AF-related complications**  **N=317** | **Asymptomatic**  **With　previous**  **AF-related complications**  **N=97** | **Symptomatic**  **No　previous**  **AF-related complications**  **N=584** | **Symptomatic**  **With previous**  **AF-related complications**  **N=76** | **P value** |
| --- | --- | --- | --- | --- | --- |
| Age (years old) | 65.8±6.4 | 67.4±7.7 | 66.0±8.8 | 69.2±7.6 | <0.001 |
| ≥ 75 years old | 27 (8.5%) | 14 (14.4%) | 94 (16.1%) | 21 (27.6%) | <0.001 |
| Women | 49 (15.5%) | 15 (15.5%) | 199 (34.1%) | 25 (32.9%) | <0.001 |
| Weight (kg) | 65.7±11.8 | 62.4±13.3 | 62.1±12.7 | ｗ | <0.001 |
| Low body weight | 43 (14.3%) | 28 (30.1%) | 125 (22.8%) | 23 (30.7%) | <0.001 |
| Non-paroxysmal AF | 168 (53.0%) | 64 (66.0%) | 90 (15.4%) | 14 (18.4%) | <0.001 |
| AF duration (years) | 1.2 (0.4-4.3) | 1.5 (0.5-5.3) | 1.4 (0.4-3.7) | 1.7 (0.5-4.7) | 0.34 |
| Hypertension | 212 (66.9%) | 71 (73.2%) | 356 (61.0%) | 53 (69.7%) | 0.04 |
| Diabetes | 61 (19.2%) | 22 (22.7%) | 99 (17.0%) | 16 (21.1%) | 0.49 |
| Previous AF-related complications | 0 (0.0%) | 97 (100%) | 0 (0.0%) | 76 (100%) | <0.001 |
| History of heart failure hospitalization | 0 (0.0%) | 37 (38.1%) | 0 (0.0%) | 76 (100%) | <0.001 |
| Ischemic stroke | 0 (0.0%) | 67 (69.1%) | 0 (0.0%) | 60 (79.0%) | <0.001 |
| CHA_2_DS_2_-VASc score | 1.8±1.2 | 3.9±1.3 | 2.0±1.3 | 4.2±1.4 | <0.001 |
| ≥ 2 | 172 (54.3%) | 95 (97.9%) | 364 (62.3%) | 75 (98.7%) | <0.002 |
| History of malignancy | 317 (18.6%) | 14 (14.4%) | 98 (16.8%) | 12 (15.8%) | 0.77 |
| eGFR (ml/min/1.73m^2^) | 52.2±16.9 | 48.5±14.0 | 55.2±17.3 | 47.4±14.9 | <0.001 |
| ≤60 ml/min/1.73m^2^ | 230 (74.7%) | 79 (83.2%) | 364 (65.0%) | 55 (75.3%) | <0.001 |
| **Echocardiographic data** |  |  |  |  |  |
| Left ventricular ejection fraction (%) | 63.1±11.0 | 59.1±15.9 | 66.4±10.0 | 64.3±12.3 | <0.001 |
| ≤ 40 % | 11 (3.9%) | 15 (16.1%) | 9 (1.6%) | 4 (5.5%) | <0.001 |
| Left atrial diameter (mm) | 43.0±8.1 | 44.5±8.8 | 39.5±7.3 | 40.0±7.6 | <0.001 |
| ≥ 50 mm | 45 (16.0%) | 23 (25.0%) | 53 (9.6%) | 7 (9.6%) | <0.001 |
| **Medications** |  |  |  |  |  |
| Oral anticoagulat | 285 (89.9) | 91 (93.8%) | 481 (82.4%) | 68 (89.5%) | <0.001 |
| Warfarin | 143 (45.1%) | 65 (67.0%) | 249 (42.6%) | 35 (46.1%) | <0.001 |
| Direct oral anticoagulants | 142 (44.8%) | 26 (26.8%) | 234 (40.1%) | 33 (43.4%) | 0.01 |
| Antiplatelet use | 59 (18.6%) | 34 (35.1%) | 106 (18.2%) | 24 (31.6%) | <0.001 |
| Anti-arrhythmic drugs | 56 (17.7%) | 20 (20.6%) | 207 (35.5%) | 31 (40.8%) | <0.001 |
| Beta blockers | 111 (35.0%) | 34 (35.1%) | 183 (31.3%) | 31 (40.8%) | 0.33 |
| Verapamil/diltiazem | 42 (13.3%) | 10 (10.3%) | 92 (15.8%) | 76 (17.1%) | 0.39 |
| Digitalis | 35 (11.0%) | 16 (16.5%) | 50 (8.6%) | 8 (10.5%) | 0.13 |
| ACEI/ARB | 142 (44.8%) | 49 (50.5%) | 222 (38.0%) | 33 (43.4%) | 0.05 |

Categorical variables are presented as number (percentage). Continuous variables are presented as mean ± SD or median and interquartile range.

AF=atrial fibrillation; BNP=brain natriuretic peptide; eGFR=estimated glomerular filtration rate; EHRA=European Heart Rhythm Association.
