# Supplementary material for: Prognostic impact of catheter ablation in patients with asymptomatic atrial fibrillation
Source: PLoS One. 2022 Dec 15;17(12):e0279178. doi: 10.1371/journal.pone.0279178 (PMC9754597; doi:10.1371/journal.pone.0279178)
Supplement: S4 Table — (DOCX) [file pone.0279178.s006.docx]

**S4 Table: Baseline characteristics comparing conservative and ablation groups in asymptomatic AF patients with and without previous AF-related complications**

| **Baseline characteristics** | **Previous AF-related complications** | | | **No Previous AF-related complications** | | |
| --- | --- | --- | --- | --- | --- | --- |
|  | **Conservative**  **group**  **N=42** | **Ablation**  **group**  **N=55** | **P value** | **Conservative**  **group**  **N=165** | **Ablation**  **group**  **N=152** | **P value** |
| Age (years old) | 67.5±8.4 | 67.4±7.3 | 0.96 | 66.0±6.5 | 65.6±6.3 | 0.53 |
| ≥ 75 years old | 7 (16.7%) | 7 (12.7%) | 0.59 | 14 (8.5%) | 13 (8.6%) | 0.98 |
| Women | 5 (11.9%) | 10 (18.2%) | 0.39 | 27 (16.4%) | 22 (14.5%) | 0.64 |
| Weight (kg) | 61.1±11.6 | 63.2±14.4 | 0.46 | 63.6±12.3 | 67.8±10.9 | 0.002 |
| Low body weight | 13 (34.2%) | 15 (27.3%) | 0.47 | 31 (21.0%) | 12 (7.9%) | 0.001 |
| Non-paroxysmal AF | 26 (61.9%) | 38 (69.1%) | 0.46 | 90 (54.5%) | 78 (51.3%) | 0.56 |
| AF duration (years) | 1.3 (0.5-4.4) | 2.1 (0.5-5.8) | 0.20 | 1.3 (0.4-4.6) | 1.0 (0.4-3.8) | 0.43 |
| Hypertension | 31 (73.8%) | 40 (72.7%) | 0.91 | 103 (62.4%) | 109 (71.7%) | 0.08 |
| Diabetes | 11 (26.2%) | 11 (20.0%) | 0.47 | 38 (23.0%) | 23 (15.1%) | 0.07 |
| Previous AF-related complications | 42 (100%) | 55 (100%) | - | 0 (0%) | 0 (0%) | - |
| History of heart failure hospitalization | 19 (45.2%) | 18 (32.7%) | 0.21 | 0 (0%) | 0 (0%) | - |
| Ischemic stroke | 25 (59.5%) | 42 (76.4%) | 0.08 | 0 (0%) | 0 (0%) | - |
| CHA_2_DS_2_-VASc score | 3.8±1.3 | 3.9±1.2 | 0.65 | 1.8±1.2 | 1.7±1.1 | 0.63 |
| ≥ 2 | 40 (95.2%) | 55 (100%) | 0.18 | 93 (56.4%) | 79 (52.0%) | 0.43 |
| History of malignancy | 7 (16.7%) | 7 (12.7%) | 0.59 | 46 (27.9%) | 13 (8.6%) | <0.001 |
| eGFR (ml/min/1.73m^2^) | 48.7±15.5 | 48.3±12.9 | 0.89 | 51.6±18.9 | 52.9±14.6 | 0.50 |
| ≤60 ml/min/1.73m^2^ | 31 (77.5%) | 48 (87.3%) | 0.21 | 109 (69.9%) | 121 (79.6%) | 0.049 |
| **Echocardiographic data** |  |  |  |  |  |  |
| Left ventricular ejection fraction (%) | 55.0±16.4 | 62.0±15.1 | 0.04 | 64.6±11.2 | 61.8±10.7 | 0.03 |
| ≤ 40 % | 8 (21.1%) | 7 (12.7%) | 0.29 | 2 (1.5%) | 9 (5.9%) | 0.046 |
| Left atrial diameter (mm) | 45.8±10.3 | 43.6±7.6 | 0.24 | 43.9±9.3 | 42.3±6.7 | 0.09 |
| ≥ 50 mm | 11 (29.7%) | 12 (21.8%) | 0.39 | 26 (20.0%) | 19 (12.5%) | 0.09 |
| **Medications** |  |  |  |  |  |  |
| Oral anticoagulat | 36 (85.6%) | 55 (100%) | 0.001 | 133 (80.6%) | 152 (100%) | <0.001 |
| Warfarin | 25 (59.5%) | 40 (72.7%) | 0.17 | 79 (47.9%) | 64 (42.1%) | 0.30 |
| Direct oral anticoagulants | 11 (26.2%) | 15 (27.3%) | 0.91 | 54 (32.7%) | 88 (57.9%) | <0.001 |
| Antiplatelet use | 14 (33.3%) | 20 (36.4%) | 0.76 | 36 (21.8%) | 23 (15.1%) | 0.12 |
| Anti-arrhythmic drugs | 5 (11.9%) | 15 (27.3%) | 0.06 | 26 (15.8%) | 30 (19.7%) | 0.35 |
| Beta blockers | 18 (42.9%) | 16 (29.1%) | 0.16 | 64 (38.8%) | 47 (30.9%) | 0.14 |
| Verapamil/diltiazem | 5 (11.9%) | 5 (9.1%) | 0.65 | 29 (17.6%) | 13 (8.6%) | 0.02 |
| Digitalis | 5 (11.9%) | 11 (20.0%) | 0.28 | 25 (15.2%) | 10 (6.6%) | 0.01 |
| ACEI/ARB | 19 (45.2%) | 30 (54.6%) | 0.36 | 72 (43.6%) | 70 (46.1%) | 0.67 |

Categorical variables are presented as number (percentage). Continuous variables are presented as mean ± SD or median and interquartile range.

AF=atrial fibrillation; BNP=brain natriuretic peptide; eGFR=estimated glomerular filtration rate; EHRA=European Heart Rhythm Association.
