# Supplementary material for: Prognostic impact of catheter ablation in patients with asymptomatic atrial fibrillation
Source: PLoS One. 2022 Dec 15;17(12):e0279178. doi: 10.1371/journal.pone.0279178 (PMC9754597; doi:10.1371/journal.pone.0279178)
Supplement: Attachment — Submitted filename: Response t o Reviewers.docx [file pone.0279178.s007.docx]

**Response to Reviewers**

**We deeply appreciate the editors and reviewers for their critically important comments and suggestions on our paper. We have revised our manuscript according to all those comments and suggestions.**

**All essential changes in the revised manuscript were highlighted in red font.**

**Our replies to the reviewers’ comments and suggestions are written below.**

**Reviewer #1: The manuscript describes the impact of catheter ablation on clinical outcomes in asymptomatic and symptomatic patients with and without previous AF complications. The manuscript was well written. However, several points need to be re-considered.

1. The major problem with this study was very complicated and confusing. Moreover, the objectives and design of the study did not match. This study aimed to explore the prognostic impact of catheter ablation in asymptomatic AF patients compared with matched AF controls. However, this study included patients with symptomatic AF. The benefits of catheter ablation for symptomatic AF have been well-discussed. If the authors discuss the benefit of the catheter ablation in asymptomatic AF patients, the patients with symptomatic AF should not be included.**

Thank you for your important suggestion. As you indicated, the main purpose of the present study was to evaluate the clinical impact of catheter ablation for asymptomatic AF. However, we also think it important to evaluate the impact in the comparison between asymptomatic and symptomatic AF patients. Indeed, in the present study, the benefit of ablation for the primary endpoint was more prominent in symptomatic AF patients than in asymptomatic AF patients. Furthermore, the superiority of ablation over conservative management in asymptomatic AF was significant only in the high-risk subset of patients with previous AF-related complications.

As you indicated, we admit that our study is a little complicated, so we have tried our best to improve the presentation style in the revised version of the manuscript to avoid confusion.

Your understanding will be deeply appreciated.

**2. On page 8, you stated that “the detailed method of the matching was described in the previous report.” However, there was no citation. I don’t know whether this matching method is reliable; can’t you use a generally accepted method such as propensity score matching?**

Thank you for the important comment. Ｗe added descriptions regarding the detailed method for matching between patients receiving catheter ablation and conservative management in the methods section, citing our previous report (Heart Vessels. 2022;37(7):1242-1254). In brief, we first selected a patient in the ablation group and attempted to find a matched control patient based on the prespecified 6 clinical risks; 1) age on the date of first AF documentation; 2) sex; 3) date of first AF documentation; 4) types of AF; **5) EHRA symptom grades (1 to 4)**; and 6) prior history of HF.

Although propensity score matching was not used, we made meticulous efforts to find out the appropriate control group of patients with conservative management. However, because we could not perfectly match the patients, there was an imbalance in the prevalence of low body weight, history of malignancy, and CKD between the ablation and conservative groups even after matching. Therefore, we performed multivariate analysis using the Cox proportional hazard model to adjust those imbalances.

We added descriptions regarding this point in the limitations section, as pasted below.

<Methods>

The current study was a post-hoc subgroup analysis of the previously reported risk-matched study.^8^ Among 4398 patients with diagnosis of AF in Kyoto University Hospital between January 2005 and March 2015, we performed 1:1 matching to find out an appropriate control patient for a given patient in the ablation group with a prespecified method as follows. First, we selected a patient in the ablation group and attempted to find a matched control patient for the selected patient in the ablation group based on the following clinical information; 1) age on the date of first AF documentation (acceptable range: ± 5 years); 2) sex; 3) date of first AF documentation (acceptable range: ± 365 days); 4) types of AF (paroxysmal or chronic); 5) European Hear Rhythm Association (EHRA) symptom grades (1 to 4); and 6) prior history of HF. When we could not find a matched control patient, the selected patient in the ablation group was excluded from the matched analysis. The control patient who had already been chosen was not matched to another patient in the ablation group to ensure 1:1 matching. Finally, we identified 1074 matched patients (537 patients in the ablation group and 537 patients in the conservative group). The detailed method of the matching was described in the previous report.^8^

In the present post-hoc subgroup study, we separately assessed the impact of catheter ablation on clinical outcomes in asymptomatic and symptomatic patients with and without previous AF-related complications (ischemic stroke or HF hospitalization).(Line 22 in Page 5 – Line 12 in Page 6)

<Limitations>

The first, the most critical limitation was the imbalance of baseline characteristics between the ablation group and the conservative group even after matching. The multivariable analyses might have not adequately eliminated the influence of unmeasured confounders in evaluating the impact of catheter ablation on the primary outcome measure. (Line 22-26 in Page 13)

**3. The ablation technique may differ since the study is from a much older period. Please describe the catheter used, mapping method, etc.**

Thank you for the suggestion. As you indicated, the duration of patient enrollment in the present study was long, 11 years. Actually, the ablation catheter used for PVI changed over time during the period. However, the therapeutic strategy of AF ablation in our center was essentially same during the period, i.e., PVI mostly by RFCA with double circular catheters along with routine tricuspid valve isthmus ablation. Cryoballoon PVI was performed only in 4.4% of patients.

We added the following descriptions in the methods section and also added S1 Table in the Supplementary materials.

<Methods>

Pulmonary veins isolation (PVI) was performed mostly by radiofrequency catheter ablation using double circular catheters, placing two 20-pollar circular-shaped catheters (Lasso, Biosense Webster or Orbiter PV, C.R. Bard Electrophysiology, Lowell, MA, USA) in ipsilateral superior and inferior pulmonary veins (S1 Table). An 8-mm tip ablation catheter (Fantasista, Japan Lifeline, Tokyo, Japan and NAVISTAER, Biosense Webster, CA, USA) was used from 2004 to 2009, and a 3.5-mm tip irrigation catheter (NAVISTAER THERMOCOOL, Biosense Webster, CA, USA) was used from 2010 to 2015. Cryoballoon (Arctic Front, Medtronic, Inc., MN, USA) was used for PVI only for paroxysmal AF since its introduction to Japan in 2014. Tricuspid valve isthmus ablation was routinely performed regardless of the presence of typical atrial flutter. Superior vena cava was isolated when it was deemed necessary. Complex fractionated atrial electrogram guided ablation was performed when sinus restoration was not obtained after PVI. Additional left atrial linear ablations were performed for sustained atrial tachycardias during the procedure.(Line 24 in Page 6 - Line 10 in Page 7)

<Supplementary material>

**S1 Table: Details of ablation procedure**

|  | **Ablation group**  **N=537** |
| --- | --- |
| Pulmonary vein isolation | 537 (100%) |
| RFCA | 513 (95.6%) |
| Cryoballoon | 24 (4.4%) |
| Superior vena cava isolation | 144 (26.8%) |
| Complex fractionated atrial electrogram guided ablation | 152 (28.3%) |
| Tricuspid valve isthmus linear ablation | 509 (94.8%) |
| Mitral valve isthmus linear ablation | 14 (2.6%) |
| Non-pulmonary vein foci ablation | 7 (1.3%) |

**RFCA=radiofrequency catheter ablation.**

**4. Further details regarding follow-up methods need to be described.**

Thank you for your important comment. Patients in the conservative group were followed mostly by the referring physicians at the local clinics without a prespecified follow-up protocol. On the other hand, most patients in the ablation group were followed with the recommended follow-up protocol at our hospital or affiliated hospitals. This difference in the follow-up methods between the groups might have influenced the study results.

We added the following descriptions in the methods and limitations sections.

<Methods>

**Ethics**

Follow-up information was obtained by review of hospital-chart and contact by letters and/or phone-call to the patient, relatives, and/or referring physicians. The follow-up protocol in the ablation groups was described in the next section. On the other hand, there were no prespecified follow-up protocol in the conservative group because the present study was a retrospective analysis. The study protocol was approved by the institutional review board in Kyoto University Hospital. Written informed consent for the catheter ablation procedure and follow-up was obtained from all patients in ablation group and we got consent for the enrollment to the study from all patients in the conservative group at the time of follow-up contact. (Line 13 - 22 in Page 6)

<Methods>

A 12-lead electrocardiogram was routinely measured at each clinical visit and 24-hour Holter monitoring was recommended at 3-, 6-, 12-month and yearly thereafter. Antiarrhythmic drug was discontinued before ablation procedure, and was restarted only when recurrent atrial tachyarrhythmias were detected. The second ablation was recommended to patients with recurrent atrial tachyarrhythmias after the blanking period of 3 months. Oral anticoagulant (OAC) was continued for at least 3 months after procedure. Thereafter, discontinuation of OAC in patients without arrhythmia recurrence was left to the discretion of the attending physician. (Line 11 - 17 in Page 7)

<Limitations>

Second, patients in the conservative group were followed mostly by the referring physicians at the local clinics without a prespecified follow-up protocol, whereas most patients in the ablation group were followed with the recommended follow-up protocol at our hospital or affiliated hospitals, which might have influenced the study results. (Line 26 in Page 13- 4 in Page 14)

**5. Please describe the ablation results and the medications used in the conservative group.**

Thank you for the important comment. According to your suggestion, we added the following Tables and Figures showing the details of ablation results and medications, as pasted below.

**S1 Table: Details of ablation procedure**

|  | **Ablation group**  **N=537** |
| --- | --- |
| Pulmonary vein isolation | 537 (100%) |
| RFCA | 513 (95.6%) |
| Cryoballoon | 24 (4.4%) |
| Superior vena cava isolation | 144 (26.8%) |
| Complex fractionated atrial electrogram guided ablation | 152 (28.3%) |
| Tricuspid valve isthmus linear ablation | 509 (94.8%) |
| Mitral valve isthmus linear ablation | 14 (2.6%) |
| Non-pulmonary vein foci ablation | 7 (1.3%) |

RFCA=radiofrequency catheter ablation.

**Table 1: Baseline characteristics comparing conservative versus ablation groups in asymptomatic and symptomatic AF patients.**

Note: Only the information regarding medications at baseline are shown here

| **Baseline characteristics** | **Asymptomatic** | | | **Symptomatic** | | |
| --- | --- | --- | --- | --- | --- | --- |
|  | **Conservative**  **group**  **N=207** | **Ablation**  **group**  **N=207** | **P value** | **Conservative**  **group**  **N=330** | **Ablation**  **group**  **N=330** | **P value** |
| **Medications** |  |  |  |  |  |  |
| Oral anticoagulant | 169 (81.6%) | 207 (100%) | <0.001 | 219 (66.4%) | 330 (100%) | <0.001 |
| Warfarin | 104 (50.2%) | 104 (50.2%) | 1.00 | 109 (33.0%) | 175 (53.0%) | <0.001 |
| DOACs | 65 (31.4%) | 103 (49.8%) | <0.001 | 112 (33.9%) | 155 (47.0%) | <0.001 |
| Antiplatelets | 50 (24.2%) | 43 (20.8%) | 0.41 | 75 (22.7%) | 55 (16.7%) | 0.049 |
| Antiarrhythmic drugs | 31 (15.0%) | 45 (21.7%) | 0.07 | 120 (36.4%) | 118 (35.8%) | 0.87 |
| Beta blockers | 82 (39.6%) | 63 (30.4%) | 0.05 | 107 (32.4%) | 107 (32.4%) | 1.00 |
| Verapamil/diltiazem | 34 (16.4%) | 18 (8.4%) | 0.02 | 67 (20.3%) | 38 (11.5%) | 0.002 |
| Digitalis | 30 (14.5%) | 21 (10.1%) | 0.18 | 32 (9.7%) | 26 (7.9%) | 0.41 |
| ACEI/ARB | 91 (44.0%) | 100 (48.3%) | 0.37 | 117 (35.5%) | 138 (41.8%) | 0.09 |

**S1 Figure: Clinical outcomes comparing asymptomatic and symptomatic AF patients in the ablation group**

A) **Event free rate from recurrent atrial tachyarrhythmias with a blanking period of 90 days after procedure**

B) **Discontinuation of OAC**

**S2 Figure: Clinical outcomes in asymptomatic AF patients of the ablation group comparing those with and without the previous AF complications**

A) **Event free rate from recurrent atrial tachyarrhythmias with a blanking period of 90 days after procedure**

B) **Discontinuation of OAC**

**Previous AF complications included the hospitalization exacerbation of HF. These patients are supposed to be symptomatically stabilized by AAD and medications for AF. Can these patients be included in the asymptomatic group?**

Thank you for your important comment. As you indicated, it may be controversial to categorize patients without any AF-related symptoms at the enrollment of the study as asymptomatic when a history of HF hospitalization was present. However, in the EHRA report (Europace 2007;9:1006–23), AF-related symptoms are recommended to be assessed at the baseline (patient enrollment). Also, importantly, we graded the symptom status at the start of the previous study (Heart Vessels. 2022;37(7):1242-1254), because symptom status was one of the prespecified 6 clinical risk factors for the matching, as written previously at the bottom of Page 2 of this reply document.

Your understanding will be deeply appreciated

**Reviewer #2: The retrospective study is interesting and reveals that both patients with symptomatic and asymptomatic AF benefit from Af catheter ablation compared to conservative treatment. A limited benefit of ablation therapy is seen in the subset of asymptomatic Af patients who did not have any previous Af-related complications.**

Thank you for your important comment on our paper. Our replies to your comments are written below.

**In addition to retrospective matched analysis, some limitations need to be mentioned and discussed :
Although, the benefit of AF ablation does not reach statistical significance in the subgroup of asymptomatic Af patients without prior complication, the log-rank p-value is 0.08. A larger prospective study needs to address this issue.**

Thank you for your valuable comments. As you indicated, the benefit of catheter ablation in asymptomatic AF patients without previous AF complications was with marginal insignificance, presumably due to the relatively small number of patients.

We added the following sentences in the limitations section.

<Limitations>

Third, this post-hoc subgroup analysis may have been underpowered especially in patients with the previous AF-related complications. (Line 4 - 5 in Page 14)

**Please provide the clinical baseline for all subgroups: As stated by the authors, the group of patients with asymptomatic AF without previous complications had more often persistent AF and were more frequently of younger age. This may imply that on a longer FU time would be necessary to detect the rare predefined major events in this younger patient group (death, hospitalisation for HF, stroke, haemorrhage). Moreover, as this group is younger, the vent rate is expected to be lower. Therefore, a higher number of patients is needed to detect statistical differences in major event rates, which are are in this young group.**

Thank you for your important comment. As you indicated, patients with previous AF complications had significantly higher cardiovascular risks and lower cardiac function relative to those without, regardless of AF symptoms. The differences in the baseline characteristics among 4 subgroups may have influenced the study results. However, the baseline characteristics of the ablation versus conservative groups were generally well balanced in all the subgroups.

According to your suggestion, we added S3 and S4 Tables, as pasted below. We also added descriptions regarding this point in the results and the limitations sections.

**S3 Table: Baseline characteristics of asymptomatic and symptomatic AF patients with and without previous AF-related complications**

|  | **Asymptomatic**  **No previous**  **AF-related complications**  **N=317** | **Asymptomatic**  **With　previous**  **AF-related complications**  **N=97** | **Symptomatic**  **No　previous**  **AF-related complications**  **N=584** | **Symptomatic**  **With previous**  **AF-related complications**  **N=76** | **P value** |
| --- | --- | --- | --- | --- | --- |
| Age (years old) | 65.8±6.4 | 67.4±7.7 | 66.0±8.8 | 69.2±7.6 | <0.001 |
| ≥ 75 years old | 27 (8.5%) | 14 (14.4%) | 94 (16.1%) | 21 (27.6%) | <0.001 |
| Women | 49 (15.5%) | 15 (15.5%) | 199 (34.1%) | 25 (32.9%) | <0.001 |
| Weight (kg) | 65.7±11.8 | 62.4±13.3 | 62.1±12.7 | ｗ | <0.001 |
| Low body weight | 43 (14.3%) | 28 (30.1%) | 125 (22.8%) | 23 (30.7%) | <0.001 |
| Non-paroxysmal AF | 168 (53.0%) | 64 (66.0%) | 90 (15.4%) | 14 (18.4%) | <0.001 |
| AF duration (years) | 1.2 (0.4-4.3) | 1.5 (0.5-5.3) | 1.4 (0.4-3.7) | 1.7 (0.5-4.7) | 0.34 |
| Hypertension | 212 (66.9%) | 71 (73.2%) | 356 (61.0%) | 53 (69.7%) | 0.04 |
| Diabetes | 61 (19.2%) | 22 (22.7%) | 99 (17.0%) | 16 (21.1%) | 0.49 |
| Previous AF-related complications | 0 (0.0%) | 97 (100%) | 0 (0.0%) | 76 (100%) | <0.001 |
| History of heart failure hospitalization | 0 (0.0%) | 37 (38.1%) | 0 (0.0%) | 76 (100%) | <0.001 |
| Ischemic stroke | 0 (0.0%) | 67 (69.1%) | 0 (0.0%) | 60 (79.0%) | <0.001 |
| CHA_2_DS_2_-VASc score | 1.8±1.2 | 3.9±1.3 | 2.0±1.3 | 4.2±1.4 | <0.001 |
| ≥ 2 | 172 (54.3%) | 95 (97.9%) | 364 (62.3%) | 75 (98.7%) | <0.002 |
| History of malignancy | 317 (18.6%) | 14 (14.4%) | 98 (16.8%) | 12 (15.8%) | 0.77 |
| eGFR (ml/min/1.73m^2^) | 52.2±16.9 | 48.5±14.0 | 55.2±17.3 | 47.4±14.9 | <0.001 |
| ≤60 ml/min/1.73m^2^ | 230 (74.7%) | 79 (83.2%) | 364 (65.0%) | 55 (75.3%) | <0.001 |
| **Echocardiographic data** |  |  |  |  |  |
| Left ventricular ejection fraction (%) | 63.1±11.0 | 59.1±15.9 | 66.4±10.0 | 64.3±12.3 | <0.001 |
| ≤ 40 % | 11 (3.9%) | 15 (16.1%) | 9 (1.6%) | 4 (5.5%) | <0.001 |
| Left atrial diameter (mm) | 43.0±8.1 | 44.5±8.8 | 39.5±7.3 | 40.0±7.6 | <0.001 |
| ≥ 50 mm | 45 (16.0%) | 23 (25.0%) | 53 (9.6%) | 7 (9.6%) | <0.001 |
| **Medications** |  |  |  |  |  |
| Oral anticoagulat | 285 (89.9) | 91 (93.8%) | 481 (82.4%) | 68 (89.5%) | <0.001 |
| Warfarin | 143 (45.1%) | 65 (67.0%) | 249 (42.6%) | 35 (46.1%) | <0.001 |
| Direct oral anticoagulants | 142 (44.8%) | 26 (26.8%) | 234 (40.1%) | 33 (43.4%) | 0.01 |
| Antiplatelet use | 59 (18.6%) | 34 (35.1%) | 106 (18.2%) | 24 (31.6%) | <0.001 |
| Anti-arrhythmic drugs | 56 (17.7%) | 20 (20.6%) | 207 (35.5%) | 31 (40.8%) | <0.001 |
| Beta blockers | 111 (35.0%) | 34 (35.1%) | 183 (31.3%) | 31 (40.8%) | 0.33 |
| Verapamil/diltiazem | 42 (13.3%) | 10 (10.3%) | 92 (15.8%) | 76 (17.1%) | 0.39 |
| Digitalis | 35 (11.0%) | 16 (16.5%) | 50 (8.6%) | 8 (10.5%) | 0.13 |
| ACEI/ARB | 142 (44.8%) | 49 (50.5%) | 222 (38.0%) | 33 (43.4%) | 0.05 |

**S4 Table: Baseline characteristics comparing conservative and ablation groups in asymptomatic AF patients with and without previous AF-related complications**

| **Baseline characteristics** | **Previous AF-related complications** | | | **No Previous AF-related complications** | | |
| --- | --- | --- | --- | --- | --- | --- |
|  | **Conservative**  **group**  **N=42** | **Ablation**  **group**  **N=55** | **P value** | **Conservative**  **group**  **N=165** | **Ablation**  **group**  **N=152** | **P value** |
| Age (years old) | 67.5±8.4 | 67.4±7.3 | 0.96 | 66.0±6.5 | 65.6±6.3 | 0.53 |
| ≥ 75 years old | 7 (16.7%) | 7 (12.7%) | 0.59 | 14 (8.5%) | 13 (8.6%) | 0.98 |
| Women | 5 (11.9%) | 10 (18.2%) | 0.39 | 27 (16.4%) | 22 (14.5%) | 0.64 |
| Weight (kg) | 61.1±11.6 | 63.2±14.4 | 0.46 | 63.6±12.3 | 67.8±10.9 | 0.002 |
| Low body weight | 13 (34.2%) | 15 (27.3%) | 0.47 | 31 (21.0%) | 12 (7.9%) | 0.001 |
| Non-paroxysmal AF | 26 (61.9%) | 38 (69.1%) | 0.46 | 90 (54.5%) | 78 (51.3%) | 0.56 |
| AF duration (years) | 1.3 (0.5-4.4) | 2.1 (0.5-5.8) | 0.20 | 1.3 (0.4-4.6) | 1.0 (0.4-3.8) | 0.43 |
| Hypertension | 31 (73.8%) | 40 (72.7%) | 0.91 | 103 (62.4%) | 109 (71.7%) | 0.08 |
| Diabetes | 11 (26.2%) | 11 (20.0%) | 0.47 | 38 (23.0%) | 23 (15.1%) | 0.07 |
| Previous AF-related complications | 42 (100%) | 55 (100%) | - | 0 (0%) | 0 (0%) | - |
| History of heart failure hospitalization | 19 (45.2%) | 18 (32.7%) | 0.21 | 0 (0%) | 0 (0%) | - |
| Ischemic stroke | 25 (59.5%) | 42 (76.4%) | 0.08 | 0 (0%) | 0 (0%) | - |
| CHA_2_DS_2_-VASc score | 3.8±1.3 | 3.9±1.2 | 0.65 | 1.8±1.2 | 1.7±1.1 | 0.63 |
| ≥ 2 | 40 (95.2%) | 55 (100%) | 0.18 | 93 (56.4%) | 79 (52.0%) | 0.43 |
| History of malignancy | 7 (16.7%) | 7 (12.7%) | 0.59 | 46 (27.9%) | 13 (8.6%) | <0.001 |
| eGFR (ml/min/1.73m^2^) | 48.7±15.5 | 48.3±12.9 | 0.89 | 51.6±18.9 | 52.9±14.6 | 0.50 |
| ≤60 ml/min/1.73m^2^ | 31 (77.5%) | 48 (87.3%) | 0.21 | 109 (69.9%) | 121 (79.6%) | 0.049 |
| **Echocardiographic data** |  |  |  |  |  |  |
| Left ventricular ejection fraction (%) | 55.0±16.4 | 62.0±15.1 | 0.04 | 64.6±11.2 | 61.8±10.7 | 0.03 |
| ≤ 40 % | 8 (21.1%) | 7 (12.7%) | 0.29 | 2 (1.5%) | 9 (5.9%) | 0.046 |
| Left atrial diameter (mm) | 45.8±10.3 | 43.6±7.6 | 0.24 | 43.9±9.3 | 42.3±6.7 | 0.09 |
| ≥ 50 mm | 11 (29.7%) | 12 (21.8%) | 0.39 | 26 (20.0%) | 19 (12.5%) | 0.09 |
| **Medications** |  |  |  |  |  |  |
| Oral anticoagulat | 36 (85.6%) | 55 (100%) | 0.001 | 133 (80.6%) | 152 (100%) | <0.001 |
| Warfarin | 25 (59.5%) | 40 (72.7%) | 0.17 | 79 (47.9%) | 64 (42.1%) | 0.30 |
| Direct oral anticoagulants | 11 (26.2%) | 15 (27.3%) | 0.91 | 54 (32.7%) | 88 (57.9%) | <0.001 |
| Antiplatelet use | 14 (33.3%) | 20 (36.4%) | 0.76 | 36 (21.8%) | 23 (15.1%) | 0.12 |
| Anti-arrhythmic drugs | 5 (11.9%) | 15 (27.3%) | 0.06 | 26 (15.8%) | 30 (19.7%) | 0.35 |
| Beta blockers | 18 (42.9%) | 16 (29.1%) | 0.16 | 64 (38.8%) | 47 (30.9%) | 0.14 |
| Verapamil/diltiazem | 5 (11.9%) | 5 (9.1%) | 0.65 | 29 (17.6%) | 13 (8.6%) | 0.02 |
| Digitalis | 5 (11.9%) | 11 (20.0%) | 0.28 | 25 (15.2%) | 10 (6.6%) | 0.01 |
| ACEI/ARB | 19 (45.2%) | 30 (54.6%) | 0.36 | 72 (43.6%) | 70 (46.1%) | 0.67 |

<Methods>

Patients with the previous AF-related complications had significantly higher cardiovascular risks and lower cardiac function than those without, regardless of AF symptoms (S3 Table). (Line 3 - 4 in Page 10)

<Limitations>

he first, the most critical limitation was the imbalance of baseline characteristics between the ablation group and the conservative group even after matching. The multivariable analyses might have not adequately eliminated the influence of unmeasured confounders in evaluating the impact of catheter ablation on the primary outcome measure. (Line 22 – 26 in Page 13)

**All four patient groups with prior AF-complications are rather small-sized patient groups ranging from 34 to 55 patients (table 2).**

Thank you for your important comment. As you indicated, the number of patients in the subgroups with previous AF complications were relatively small, as shown in S3 and S4 Tables shown above.

We added descriptions regarding this point in the limitations section.

Third, this post-hoc subgroup analysis may have been underpowered especially in patients with the previous AF-related complications. (Line 4 - 5 in Page 14)
